# The Growth of Hospital Expenditure Considered in Relation to Greater London

**Published:** 1906-04-07

**Authors:** 


### HOSPITAL ADMINISTRATION.

### CONSTRUCTION AND ECONOMICS.

### THE GROWTH OF HOSPITAL EXPENDITURE CONSIDERED IN RELATION TO GREATER LONDON.

In continuation and completion of the article on "The Resources and Requirements of the Voluntary Hospitals of London," which we published in our issue of February 11, 1905, we now print a paper which deals exhaustively with the hospitals and the needs of the population in Greater London. It is taken from the first chapter of the 1906 edition of Burdett's "Hospitals and Charities."

Last year we gave a ten years' review of the expenditure of the London hospitals and its lessons. The conclusions derived from the figures which we then gave, and which dealt with the growth in the number of patients treated at and the revenue and expenditure of the hospitals in the administrative county of London compared with the growth in the population of that area during the ten years ended with 1903, were the following :—

1. That the population of the administrative county of London had only increased 6 per cent. in the ten years, whilst the percentage of increase in the hospital population was 23.21 per cent.

2. That the yearly income, including legacies, of the 94 voluntary hospitals in the administrative county of London had increased in the ten years by £272,000, and that the total amount of money contributed to these hospitals in 1903 was £1,331,090, as compared with £807,785 in 1894, an increase of nearly £525,000. That is to say, the amount of money contributed to these hospitals was nearly 65 per cent. more in 1903 than it had been in 1894.

3. That the total expenditure of these 94 voluntary hospitals in the administrative county of London in 1894 was £837,725 (namely, ordinary £703,635 and extraordinary £134,090), whilst in 1903 it was £1,341,641 (namely, ordinary £914,373 and extraordinary £427,268), or an increase of no less than £503,916, equivalent to 60 per cent.

4. That it was obvious on the face of it that this huge increase in the expenditure of the 94 voluntary hospitals could not be justified by the extra number of patients treated, namely, that the increased work did not account for the increased expenditure; and

5. We held strongly that the hospitals owed it to their reputation and to their acceptance of the grants to them by the King's Fund to make an effort to keep their expenditure within their income now that this income should be in fact adequate to meet every legitimate claim upon it by those patients who were really entitled to receive free medical relief.

#### An Extension of the Inquiry to the Outer Ring.

In dealing with the matter last year we purposely confined our statement to the administrative county of London and the 94 voluntary hospitals situated therein because it would not have been possible to find space adequately to treat all the figures and facts connected with the extension of the inquiry to the "Outer Ring" and contiguous populations. We now, however, propose to extend our inquiries to the "Outer Ring," and a study of the figures will convince most thoughtful people that although the population of the administrative county of London during the decade 1894 to 1903 only increased by 6 per cent., whilst the population of the "Outer Ring" increased in the same period by 45 per cent., yet this does not diminish, but rather increases, the force of the criticisms which we made last year in regard to the expenditure of the 94 voluntary hospitals in the county and the increase in the growth of the hospital population.

There are, of course, some who consider that any stick is good enough to beat a dog with, and that a great question like the medical relief of poorer Londoners can be disposed of in a slap-dash manner by the production of a few percentages, without any examination or test of the figures and their bearing, or the meaning which properly attaches to the increases dealt with.

#### The Extent of the "Outer Ring."

In order to understand the bearing of the "Outer Ring" and its population upon the volume of hospital relief provided each year by the 94 voluntary hospitals in the county of London, it is necessary in the first instance to bring out clearly what localities and communities are included in the "Outer Ring." Possibly as good a description of the "Outer Ring" as any that has been given is that which appears in the report of the Royal Commission on London Traffic, which defines the "Outer Ring" as "containing every parish, the whole of which is within fifteen miles of Charing Cross, or any portion of which is within twelve miles, and it contains 692.84 square miles, including land and inland water, but excluding tidal water and foreshore. Within this area of 692.84 square miles there are six administrative county authorities—namely, those of London, Middlesex, Surrey, Kent, Essex, and Hertford." This vast area therefore covers nearly 700 square miles, and it is important, too, to remember that, taking Charing Cross as a centre, it stretches seventeen miles north to Cheshunt, nineteen miles south to Banstead Heath, fourteen miles east to Dartford, and eighteen miles west to Staines.

#### How Far do Out-patients from the "Outer Ring" Attend London Hospitals?

The next point to remember is that of the 1,760,640 patients relieved by the 94 voluntary hospitals in the county of London for 1903, 1,648,353, or 93.6 per cent., were out-patients. It may be taken for granted also that very few of the out-patients relieved by these hospitals took the trouble to travel nineteen miles, or even fourteen miles, once or twice a week, in order to get a bottle of medicine at a London hospital. Why should they indeed, seeing, as we shall show directly, that the outlying districts have, as a matter of fact, good hospital accommodation, which is rapidly being extended in response to the growth of the population in certain areas?

In order to fairly consider this question from all points of view, it is necessary to point out that on the east side the distance of the boundary of the "Outer Ring" from Charing Cross is much less than it is on the north, south, and west sides of the area in question. It follows that the residents of the "Outer Ring" on the East are brought into closer touch with those Metropolitan hospitals which minister to the requirements of that dense working population which resides within, comparatively, a short distance of the London and Poplar hospitals. No doubt these latter hospitals are resorted to by some of the residents in the "Outer Ring," although every year the West Ham Hospital and other local hospitals are being developed, and the volume of medical relief which they give enhances their popularity, and tends to make them more and more the institution to which the inhabitants of these districts look for aid when overtaken by accident or disease. The figures we give in the table which follows in regard to the voluntary hospitals in the "Outer Ring" confirm this view.

Apart, however, from the use made of the London and the Poplar hospitals by residents of the "Outer Ring" on the east side of the boundary of the county of London, the circumstances (1) that the population on the north, south and west sides reside too far away from the hospitals in the administrative county of London to make it possible for them to use their out-patients' departments habitually, and (2) that the residents in those districts to a large extent consist of comparatively well-to-do people who do not need, and ought not, in fact, to receive, free medical relief, renders it probable that for all practical purposes, with the exception of the east side of the boundary, few, if any, of the residents in the "Outer Ring" seek out-patient relief at any of the 94 voluntary hospitals in the county of London which we included in our review last year.

### In-patients Resident in Outer Ring.

Further, as the out-patients formed 93 per cent. of the total number of the patients relieved in 1903 at the 94 voluntary hospitals in the county of London, so for practical purposes the number of residents in the "Outer Ring" who may decide to pass by the excellent hospitals situated in their own neighbourhood, and to go to a London hospital, are necessarily but a drop in the ocean of relief, seeing that they can form but a very small portion of the hospital population relieved as in-patients in the hospitals of London, the whole number of which are under 7 per cent. of the total patients relieved.

This very circumstance, coupled with the fact that the population in the "Outer Ring" increased by 45.3 per cent. whilst that of the administrative county of London only increased by 7 per cent. in the last census decade, 1891-1901, enforces the conclusion we arrived at, that the amount of free medical relief at present rendered by the 94 voluntary hospitals in question is excessive, and far beyond the needs and requirements of those members of the population who can properly claim it as a free gift from a public institution.

### Is the Free Medical Relief Excessive?

So far, then, our examination of the circumstances tends to show that, when the "Outer Ring" is included in our purview the evidence of excessive free relief by the hospitals in the county of London increases in force, and that the supposed shifting of the population from the "Inner" to the "Outer Ring," if it exists at all to any appreciable extent at the present time (which we beg leave to doubt), should make every conscientious hospital manager apprehensive, and force him to face this question of increasing expenditure, with a view to reducing it within reasonable limits. In support of this latter view an analysis of the increase and decrease of the population in "Inner" London and the "Outer Ring" reveals the fact that, with the exception of the City itself, most of the boroughs in the central area had a smaller percentage of increase in their population in the decade ending 1901 than they had in the decade ending 1891. In the central area of the administrative county of London, including the City, in 1901 there was a decrease in population of 3.4 per cent., as compared with a decrease of 4.1 per cent. in the previous decade ending 1891. In the remaining portion of "Inner" London the increased percentage in the population for the decade ending 1901 was only 12.8 per cent., compared with an increase of 19.7 per cent. in the previous ten years. So also in regard to the population of the "Outer Ring," where the increased percentage in the population in the decade ended 1901 was only 45.3 per cent., compared with an increase of upwards of 50 per cent. in each of the three previous decades. These figures, as the Registrar-General points out, make it appear that the overflow of the Metropolitan population may now extend, and is, in fact, extending, even beyond Greater London. It follows from these figures and the latter conclusion that the managers of the 94 voluntary hospitals will soon have to consider, if they are not already considering, how far, if at all, it is justifiable to go on erecting new and expensive hospital buildings in the administrative county of London, seeing that the population formerly served by those hospitals is being removed farther and farther away from their locality, and that in consequence it is becoming more and more difficult for an ever-increasing number of the population they used to serve to seek out-patient relief, at any rate, at their institutions.

### Beds and Patients at the Hospitals in (a) the County of London and (b) the "Outer Ring."

Next let us consider how far the increase in the growth of the population of the "Outer Ring" has been met by the hospitals which are situated in this area itself. The following table supplies the answer and shows at a glance the hospital beds, and the patients treated in (a) the administrative county of London; and (b) in the "Outer Ring":—

Table I.—Showing the Number of Beds and the Number of In- and Out-patients in the Hospitals in (a) the County of London and (b) the "Outer Ring" During the Years 1894 and 1903.

| | 1894. | | 1903. | | Percentage Increase 1903 over 1894. |
|---|---|---|---|---|---|
| Beds— | | | | | |
| County of London . | 9,110 | | 9,771 | | 7.25 |
| "Outer Ring". . | 546 | | 834 | | 52.74 |
| | | 9,656 | | 10,605 | 9.83 |
| In-Patients— | | | | | |
| County of London . | 92,515 | | 112,287 | | 21.37 |
| "Outer Ring". . | 4,369 | | 9,344 | | 113.87 |
| | | 96,884 | | 121,631 | 25.54 |
| Out-Patients— | | | | | |
| County of London . | 1,336,416 | | 1,648,353 | | 23.34 |
| "Outer Ring". . | 40,384 | | 81,206 | | 101.08 |
| | | 1,376,800 | | 1,729,559 | 25.62 |
| Grand Total of Patients | | 1,473,684 | | 1,851,190 | 25.61 |

It will be seen that, whereas the number of hospital beds provided in the county of London only increased during the decade ending 1903 by 7.25 per cent., the number of hospital beds provided in the "Outer Ring" increased in the same period by 53 per cent. The in-patients relieved by the hospitals of the county of London increased in the decade by 21 per cent., but in the "Outer Ring" they increased by 114 per cent. Again, the out-patients relieved by the hospitals in the county of London increased in the ten years by 23 per cent., whilst the out-patients relieved by the hospitals in the "Outer Ring" increased by 101 per cent. No useful purpose can be served by labouring these figures, which must convince any searcher after truth that the whole condition of medical relief in the county of London, so far as the voluntary hospitals are concerned, requires reconsideration and reconstruction.

### Beds and Patients at the Hospitals in the "Outer Ring."

In support of the views we have ventured to put forward on this subject, we have thought it desirable to give a table of the hospitals of various sizes and importance which are situated in the "Outer Ring," which follows. We have given in each case the number of beds and the in- and out-patients treated in the years 1894 and 1903, as they add to the interest of the table and strongly support the general conclusions we have ventured to draw. It will be noticed that the table affords evidence that where the demands of the population by its growth have called for further hospital accommodation such accommodation is gradually being supplied. At Tottenham, for instance, the local hospital is being largely extended, and will no doubt shortly contain at least 200 beds.

Table II.—Table Showing the Number of Beds and Patients Treated at the Hospitals in the "Outer Ring" in the Years 1894 and 1903.

| | 1894. | | | 1903. | | |
|---|---|---|---|---|---|---|
| | Beds: | In-Patients. | Out-Patients | Beds. | In-Patients. | Out-Patients |
| West Ham Hospital ... ... ... ... ... | 57 | 272 | 17,088 | 60 | 1,036 | 23,319 |
| St. Mary's Hospital, Plaistow ... ... ... ... | 22 | 46 | ... | 35 | 588 | 15,114 |
| Leyton, Walthamstow, etc., General Hospital ... | 20* | 221 | 100 | 45 | 580 | 2,712 |
| Woodford Jubilee Hospital ... ... ... ... | ... | ... | ... | 15 | 126 | — |
| Tottenham Hospital ... ... ... ... .. | 70 | 771 | 9,703 | 73‡ | 953 | 18.870 |
| Enfield Cottage Hospital ... ... ... ... | 9 | 73 | ... | 14 | 169 | — |
| Wood Green Cottage Hospital ... ... ... ... | ... | ... | ... | 25 | 368 | — |
| Cheshunt Cottage Hospital ... ... ... ... | 8 | 54 | ... | 8 | 53 | — |
| Potter's Bar Cottage Hospital ... ... ... ... | 10 | 85 | 29 | 11 | 83 | — |
| Barnet Cottage Hospital ... ... ... ... | 16 | 144 | ... | 22 | 229 | — |
| Stanmore Cottage Hospital ... ... ... ... | ... | ... | ... | 8 | 75 | 132 |
| Harrow Cottage Hospital ... ... ... .. | 12 | 58 | ... | 12 | 138 | 40 |
| Bushey Heath Cottage Hospital ... ... ... | ... | ... | ... | 20 | 115 | — |
| Acton Cottage Hospital ... ... ... ... ... | ... | ... | ... | 24 | 132 | 1,061 |
| Hayes Cottage Hospital... ... ... ... ... | 6 | 15 | ... | 6 | 19 | — |
| Hillingdon Cottage Hospital ... ... ... ... | 6 | 41 | ... | 6 | 36 | — |
| Hanwell Cottage Hospital ... ... ... ... | ... | ... | ... | 7 | 46 | 33 |
| Hounslow Cottage Hospital ... ... ... ... | 10 | 77 | 941 | 16 | 166 | 966 |
| Twickenham, St. John's Hospital ... ... ... | 14 | 142 | ... | 14 | 143 | 296 |
| Teddington Cottage Hospital ... ... ... ... | 14 | 128 | ... | 22 | 188 | 220 |
| Richmond, Royal Hospital ... ... ... ... | 40 | 383 | 3,119 | 60 | 660 | 4,414 |
| Kingston-on-Thames, Victoria Hospital ... ... | ... | ... | ... | 12 | 178 | — |
| Thames Ditton Cottage Hospital ... ... ... | 5 | 33 | ... | 10 | 100 | — |
| Epsom and Ewell Cottage Hospital ... ... ... | 16 | 115 | ... | 16 | 106 | — |
| Wimbledon Cottage Hospital ... ... ... ... | 11 | 99 | ... | 16 | 156 | 36 |
| South Wimbledon Cottage Hospital ... ... ... | ... | ... | ... | 12 | 165 | 118 |
| Surbiton Cottage Hospital ... ... ... ... | 19 | 220 | ... | 19 | 181 | — |
| Carshalton Cottage Hospital ... ... ... ... | ... | ... | ... | 7 | 82 | — |
| Sutton Hospital ... ... ... ... ... ... | ... | ... | ... | 14 | 115 | — |
| Beckenham Cottage Hospital ... ... ... ... | 20 | 100 | ... | 32 | 239 | 301 |
| Croydon General Hospital ... ... ... ... | 80 | 665 | 8,798 | 95 | 1,021 | 12,988 |
| Bromley Cottage Hospital ... ... ... ... | 20 | 189 | ... | 24 | 399 | — |
| Bromley, Phillips Memorial Hospital ... ... ... | 10 | 75 | 525 | 11 | 136 | 430 |
| Bexley Cottage Hospital ... ... ... ... | 9 | 52 | ... | 9 | 46 | 29 |
| Sidcup Cottage Hospital ... ... ... ... | 12 | 99 | 50 | 12 | 144 | 57 |
| Chiselhurst, Cray, etc., Cottage Hospital ... ... | 14† | 86 | ... | 21 | 222 | — |
| Buckhurst Hill Village Hospital ... ... ... | 6 | 57 | ... | 8 | 66 | — |
| Erith Cottage Hospital ... ... ... ... ... | 10 | 69 | 31 | 13 | 77 | 50 |
| | 546 | 4,369 | 40,384 | 834 | 9,344 | 81,206 |

* Figures for 1895. † Figures for 1892. ‡ 40 extra beds are being added to the Hospital.

### Ill-founded Criticisms Refuted.

In conclusion we venture to point out that the argument that because the population of the "Outer Ring" has increased 45 per cent. in the ten years 1894 to 1903, therefore the hospitals in the county of London have had to meet the extra work entailed by that increase, is shown by the foregoing tables and figures to be unsound in substance and in fact. To advance an argument of this kind is to display a want of perception and grasp which no hospital manager who values his reputation would surely care to reveal. There are two facts which at once prove how ill-founded such an argument is. One is that the hospital patients relieved by the hospitals situated in the "Outer Ring" have increased by 103 per cent., or almost in the same proportion to the increase in the population, as we showed last year was the case in connection with the county of London—which demolishes the argument. The second is that, if the whole area comprised in the "Outer Ring" is to be taken as the area served by the hospitals in the county of London, then there must be added to the 94 hospitals in the county all the other hospitals situated within the area outside the county, and we must compare the growth of the population of the whole area and not base our argument on the growth of the population of only a portion of the district. The population of the whole area of Greater London (namely, the administrative county and the "Outer Ring") has increased in the ten years ending 1903 from 5,964,443 to 6,806,296, an increase of 14 per cent. in the decade, whilst the patients treated at all the hospitals situated within the whole area have increased 25½ per cent. These figures show that the voluntary hospitals in Greater London have actually undertaken 81 per cent. more work than the increase in the population of the district warranted. This application to our conclusions of a test proposed by one of our critics—a test which he proudly declared upset our argument—effectually exposes the fallacy of contending that the arguments based upon our ten years' review will not bear the test of the closest examination. It will be seen that the exact contrary is the case.

But apart from this, and any argument which may be used against the conclusions drawn from the growth in the number of patients relieved, compared with the increase in the population, our main contention must not be allowed to sink into oblivion. That contention was, and is, that there had been an inexplicable increase in the expenditure of the 94 voluntary hospitals during the ten years ended 1903, and that this increase was out of all proportion to the increased needs and requirements of the poorer population of even Greater London. This is a most material fact, which the managers of the 94 voluntary hospitals have to face. It must, indeed, be altered, too, if the voluntary system of hospital support is to maintain its reputation, and to continue to be the glory and the pride of our nation and empire.

